# β-Hydroxybutyrate Regulates Activated Microglia to Alleviate Neurodegenerative Processes in Neurological Diseases: A Scoping Review

**DOI:** 10.3390/nu15030524

**Published:** 2023-01-19

**Authors:** Siva Shantini Jayashankar, Khaizurin Tajul Arifin, Muhammad Luqman Nasaruddin

**Affiliations:** Department of Biochemistry, Faculty of Medicine, Universiti Kebangsaan Malaysia Medical Centre (UKM-MC), Kuala Lumpur 56000, Malaysia

**Keywords:** beta-hydroxybutyrate, microglia, neurodegenerative disease, inflammation, ketogenic diet

## Abstract

This scoping review aimed to summarise the effects of the ketone body β-hydroxybutyrate. The review details the revealed pathways and functional properties following its intervention in the context of neurodegenerative diseases. In this study, 5 research publications that met the inclusion and exclusion criteria were shortlisted. Following the intervention, we discovered a tendency of reduced inflammatory status in microglia, as evidenced by lower levels of pro-inflammatory mediators produced, reduced microgliosis in afflicted tissues, and enhanced cognitive functions in neurodegenerative models. We found that there is a significant overlap in the mechanism of action of β-hydroxybutyrate (BHB) via activation of the G-protein-Coupled Receptor 109A (GPR109a) receptor and deactivation of the inflammasome complex. Furthermore, although comparing outcomes can be challenging due to the heterogeneity in the study model, the results we have assembled here were consistent, giving us confidence in the intervention’s efficacy. We also discussed new studies where BHB is involved in various roles in regulating inflammation in microglia, allowing for fresh therapeutic targets against neurodegeneration. This brief review provides evidence to support the huge potential of BHB in the treatment of neurodegenerative illnesses.

## 1. Introduction

The numerous breakthroughs in medical research and social education allow for the extension of life expectancies seen in the older population today. According to recent reports, the component of people 65 and older is anticipated to nearly double by 2050, rising from 703 million in 2019 to a staggering 1.5 billion by 2050 [1]. Eastern and southern Asia, Latin America, and the Caribbean are estimated to experience economies with old-age dependency ratios similar to those currently seen in northern and eastern European countries, which explains why the pace of population aging is much more rapid in these regions [1].

While longevity is one of the most celebrated modern scientific achievements, it does not necessarily translate into improved healthier living. With advancing age, deleterious changes in the body system are typically associated with deterioration of functionality and increased vulnerability to death, which reduces the overall quality of life [2,3,4,5,6,7,8]. This has led to the shift of focus of public policies from prolonging life to improving overall health span [9,10,11,12,13]. Numerous interventions targeting lifestyle changes have been studied to promote healthy aging [14,15,16]. Dietary interventions in particular have been found to improve age-related conditions and maintain the functional status of aged individuals [17,18,19,20,21]. Dietary patterns such as the Nordic Diet, Mediterranean Diet, and several variations of the Asian diet have been shown to alleviate inflammatory processes caused by aging and thus promote a healthier way of living [22].

Additionally, it has been demonstrated that the ketogenic diet (KD) has comparable health advantages [23,24,25,26]. This low-sugar, moderate-protein, high-fat diet was recently discovered to significantly increase the life expectancy of aged mice while maintaining their physiological functions in comparison to controls [23]. As it mimicked caloric restriction, it presented similar health outcomes in terms of disease improvement through increased ketone body production [24,27]. These molecular breakdown products of ketones are typically known as substitutes for fuel under glucose-deprived conditions [28]. However, interestingly, it was recently reported that these ketone bodies played an active role in regulating inflammation [29,30,31,32,33].

Chronic aberrant inflammation is a common component of all neurodegenerative illnesses [34]. As there is no definitive cure for these multifactorial disorders, patients have to rely on drugs and other therapeutic management for symptomatic relief. Although inflammation is a direct defense mechanism that protects brain tissues, the sustained inflammatory responses observed in neurodegenerative diseases exacerbate the healing processes and become one of the main causes that further aggravate these neurological conditions. Inflammation in the central nervous system is tightly regulated by microglia and astrocytes [35]. Traditionally, microglia undergo two different responsive pathways, depending on the stimuli [36]. The M1 phenotype is responsible for the immune response and the release of proinflammatory mediators, whereas the M2 phenotype is activated for its anti-inflammatory properties [36,37]. It was recently suggested that the manipulation of the microglial phenotype could potentially be used as a possible option for therapeutic intervention against neurological disorders [38,39].

The prospective application of β-hydroxybutyrate (BHB) in the study of inflammation, especially in microglia, is gaining traction steadily. In lipopolysaccharide (LPS)-activated microglial cells, a recent study demonstrated that BHB can control metabolic reprogramming by raising the expression of essential proinflammatory markers [40]. Others have reported ramifications towards the M2 phenotype in vitro and in vivo following BHB treatment [41,42]. The potential protective and preventive properties of BHB are substantial considering the direct modulatory effects it exerts on individual microglia. We hope to see more published research on the observable pathways and functional properties that change after treatment in activated microglia. The impact of this ketone body on microglial activity in neurodegenerative disorders is still a subject of much research. We hope that this scoping review will provide a succinct explanation of how BHB behaves whilst uncovering the gaps in the literature. In addition, we hope to provide relevant recommendations for future considerations.

## 2. Materials and Methods

A systematic search for peer-reviewed English articles dated from January 2005 to August 2022 has been conducted through the databases: Scopus, Web of Science (WOS), and PubMed. Keywords together with the Boolean operator search words AND and OR were used to map pertinent articles for the current study. The search string included “BHB” OR “BHBA” OR “Beta-hydroxybutyrate” OR “Beta-hydroxybutyric acid” AND “microglia”. The inclusion criteria were (1) a study that explains the responses of the inflammatory pathways or the signaling responses of BHB’s influence on microglia, (2) an in vivo and in vitro study using animal models, animal microglial cell lines, and humans, (3) a study focused only on neurodegenerative disorders, and (4) studies that focused only on BHB ketone body. The exclusion criteria consist of (1) studies that focus on other ketone bodies such as acetone or acetoacetate, and (2) studies that discuss other biochemical pathways not involved in microglial inflammatory responses. All articles were reviewed and rated for eligibility with the contribution of all authors.

## 3. Results

A total of 77 articles were found in the literature search conducted on 6 September 2022, according to Scopus (25), Web of Science (WOS) (33), and PubMed (19). Based only on the abstract and title, 58 articles were eliminated after being determined to be duplicated. The eligibility of the 16 remaining articles was carefully examined. Five original research articles were found to meet the criteria and were chosen for review. Due to the scope of the current investigation, 11 papers were eliminated since they were general studies on neuro-inflammation rather than those that are focused on neurodegenerative diseases. The interventional treatment given to the animals was a combination of ketone bodies via the ketogenic diet route instead of BHB alone, even if these articles do examine inflammatory profiles. The crux of the current review paper is made up of the 5 nominated research as they examined the potential mechanisms that BHB modifies in especially activated microglia. The summary of the data extracted for this review is provided in the Table 1.

### 3.1. Neurodegenerative Diseases

#### 3.1.1. Alzheimer’s Disease (AD)

Two articles were chosen that described the effects of BHB on microglia in the context of Alzheimer’s disease. Both investigations used the 5XFAD mouse model, a preclinical transgenic model that overexpresses mutant human amyloid beta (A4) precursor protein 695 (APP) with the Swedish (K670N, M671L), Florida (I716V), and London (V717I) familial Alzheimer’s disease (FAD) mutations, as well as two FAD mutations, M146L and L286V of the human presenilin 1 [46]. As these mice were similar in age at the time of the study, comparable results were possible. These mice were given BHB in two different ways: in one study, it was dissolved in the mice’s drinking water [43], and in the other, it was injected subcutaneously [44]. Furthermore, the duration of the intervention varied, with one study lasting 3 weeks [44] and another lasting 2 months [43]. However, one study focused on incorporating a single gender (female) for their experiment [44], while the other used both (males and females) [43]. Both studies found a significant reduction in plaque number and percent area covered by these plaques after BHB treatment when compared to untreated control mice. Microgliosis was also altered in BHB-treated mice compared to controls. These studies also identified a decline in primed proinflammatory microglia, which was followed by a decline in the quantity of pro-inflammatory mediators released in the cortex and/or hippocampus. Furthermore, BHB treatment reduced NLRP3 inflammasome activation in these mice’s cortices when compared to controls. Interestingly, compared to controls, BHB treatment increased levels of NEP, a β-amyloid degradation enzyme, and ACE, a peptidase that degrades βA-40 and βA-42 in a GPR109A-dependent manner in these mice. More importantly, in these mice, BHB improves cognitive impairment.

#### 3.1.2. Parkinson’s Disease (PD)

Two articles describing the effects of BHB on microglia were chosen. Both papers used in vitro research [33,45], but only one employed an animal model; LPS was stereotaxically administered to male Wistarw rats in order to elicit or mimic neuropathological markers and inflammatory processes in clinical Parkinson’s disease, by selectively inducing dopaminergic neuron death in nigrostriatal system, microglial overactivation and pro-inflammatory mediator release [33]. Similarly to AD, BHB treatment in the PD model demonstrated inhibition of microglial activation and decreased mRNA expression of proinflammatory mediators. Furthermore, after LPS activation, GPR109A expression was found to increase in primary microglial cultures. This activation was inhibited, particularly in BHB-pretreated cells, resulting in a reduction of pro-inflammatory cytokine levels release that is GPR109A dependent. Furthermore, a decrease in the p65 protein was observed in pretreated cells, which implies that Nf-kB activation is negatively regulated by the same GPR109A pathway. Interestingly, BHB treatment does not prevent fibrillar synuclein aggregate-induced inflammasome activation in primary microglial cells, unlike ATP and monosodium urate (MSU). Although this is the case, behavioral improvements is detected in BHB-treated rats [44].

#### 3.1.3. Spinal Cord Injury (SCI)

In the current review, only one animal study was considered for this disease. In SCI-model rats, BHB administration reduced the levels of proinflammatory mediator proteins. Additional in vitro studies showed that BHB inhibited NLRP3 inflammasome activation (via ATP) in BV2 cells, promoting the polarisation of microglia from the M1 to M2 phenotype.

## 4. Discussion

This scoping review’s main goal is to evaluate the evidence for BHB’s possible preventive implications on the microglial functions in neurodegenerative disorders. The original articles that were shortlisted highlight BHB’s ability to reduce proinflammatory cytokine levels and promote the polarization of activated microglial cells to a resting phenotype in both in vitro and/or in vivo models of AD, PD, or SCI.

### 4.1. BHB, and G-Coupled Protein Receptor 109A (GPR109A)/Hydrocarboxylic Acid Receptor 2 (HCAR2)

The inhibitory action of BHB on microglia depends on its engagement with the functional receptor, GPR109A. G-coupled protein receptor 109A (GPR109A) was discovered to be a niacin receptor [47]. It is present in a wide range of tissues and cell types and is mostly found in cellular membranes [48]. Later, it was discovered that BHB could also activate this receptor and inhibit lipolysis in mouse adipocytes at fasting serum concentrations, similar to niacin [49]. This then led to a surge of new research on the dynamics of BHB in GPR109A.

Manipulation of GPR109A levels would have a significant impact on BHB’s ability to regulate the inflammatory actions of microglia. GPR109A expression levels are increased in tissues and cells following pathological insults, according to recent findings [33,44]. In contrast to wild-type, GPR109A expression was found to be higher in the brains of transgenic AD models and in primary microglial cultures stimulated with LPS in a dose- and time-dependent manner [33,44]. The increased expression levels observed are thought to indicate a negative feedback loop that limits excessive inflammation [44]. Furthermore, BHB was discovered to regulate the expression of GRP109A. In a different experiment, the researchers showed that mice that were inducibly expressing UNG, a mutant form of the mitochondrial DNA repair enzyme, showed a similar rise in GPR109A expression following a keto-based diet as compared to the wild-type and standard diet mice [50].

Knocking down GPR109A in LPS-activated primary microglial cells [33] or blocking the receptor with PTX toxins in BV2 cells [51], abrogated the neuroprotective effects of BHB-pretreatment on lowering the pro-inflammatory mediators release, and downregulation of the NF-kB. Furthermore, in the context of AD, blocking or knocking down GPR109A abolished the regulation of NEP and APP expression by BHB in 5XFAD brains [44]. The activation of GPR109A receptors by BHB suppresses proinflammatory signaling pathways and the production of proinflammatory mediators.

### 4.2. BHB and Node-Like-Receptor-Family Pyrin Domain Containing 3 (NLRP3) Inflammasome

The NLRP3 inflammasome is assembled and activated when the NLRP3 intracellular sensor recognizes a variety of pathogenic/damage-associated molecular patterns (PAMP/DAMPs). Pro-inflammatory cytokines like *IL-1β* and IL-18 are released as a result of the formation of the inflammasome, which is dependent on the ASC (apoptosis-associated speck-like protein containing a caspase recruitment domain (CARD)) adaptor and caspase-1 effector [52]. Following NLRP3 activation, ASC is recruited and forms a large protein complex (speck), which then recruits caspase-1, allowing it to self-cleave and activate, resulting in the release of downstream pro-inflammatory cytokines [52].

Recent findings have suggested the critical role of the inflammasome in the progression of neuroinflammation in neurodegenerative disorders. ASC speck is thought to act as a scaffold for the growth and spread of misfolded protein aggregates. At least in AD, a significant amount of ASC with distinct pattern recognition receptors was found in microglia and astrocytes associated with β-amyloid in the hippocampus of old-aged AD mice [53]. It was reported that the pro-inflammatory response is enhanced by ASC-β-amyloid composites, which causes pyroptotic cell death of microglia that further releases functional ASC, and thus creates a vicious cycle [54]. It was demonstrated that an AD mouse model injected intrahippocampally with ASC specks, showed seeding and spreading of the β-amyloid pathology in the brain region [55]. In contrast, the homogenates from the brains of the AD mouse model failed to exert the same observation in ASC-deficient AD mice [55]. Additionally, it was discovered in a different study that Tau-seeding reduced microgliosis in Tau mice lacking ASC. When compared to ASC-Tau mice, IBA-1 staining following Tau seeding was significantly reduced in ASC-deficient Tau mice [56]. In a different study, it was discovered that tau hyperphosphorylation was decreased in the granular cell layer of the dentate gyrus, the CA1 cell body area, and the hippocampus [57]. Another study found similar results, with significantly less hippocampal atrophy in mice after tau [58]. Cleaved caspase-1 and ASC were found to be significantly more abundant in PD patients’ brains than in controls [59]. In the same study, fibrillar α-synuclein activated NLRP3 in mouse microglia, which led to a delayed but significant activation of the NLRP3 inflammasome and the release of extracellular *IL-1β* and ASC [59]. Moreover, another study showed that nigral-dopaminergic degeneration and pathological α-synuclein are prevented in NLRP3-knockout PD mice [60].

KD anti-inflammatory effects are also linked to BHB-mediated inhibition of the NLRP3 inflammasome. The ability to control its deactivation and/or identify the inhibitory pathways associated with NLRP3 opens therapeutic possibilities for slowing the progression of neurodegenerative diseases. According to our review, at least one study has demonstrated that BHB lessens AD pathology by preventing NLRP3 activation [43]. Exogenous BHB administration reduces ASC-speck formation and activation, as well as mature caspase-1, lowering the amount of *Il-1β* secreted in transgenic AD mice [43]. The results are in line with those of an earlier study in which AD mice lacking NLRP3 or caspase-1 showed reduced brain levels of caspase-1 and *Il-1β* [61]. Additionally, in SCI, it was also reported that NLRP3 expression was significantly reduced by the KD after injury [42]. In comparison to the standard diet group, the KD group had lower expression of ASC and caspase-1 p20 in spinal cord tissue [42]. BHB is thought to control an unknown upstream event that reduces K+ efflux from macrophages as well as by inhibiting ASC downstream activities to block the NLRP3 inflammasome-mediated inflammatory disease [62]. It is important to note that BHB inhibition of the NLRP3 is independent of the GPR109a, indicating that BHB has broad effects and might simultaneously modulate several pathways [62].

Furthermore, it was discovered that BHB could inhibit ATP and monosodium urate (MSU)-induced inflammasome activation, which was consistent with the findings of the AD, PD, and SCI studies reviewed here [42,43,45]. However, unlike these two activators, BHB did not prevent the release of *IL-1β* or caspase-1 or the activation of the inflammasome caused by α-synuclein fibrils. This suggests that the upstream events modulated by BHB are not necessary for the inflammasome to be activated by α-synuclein fibrils [45]. The inhibitory effect of BHB on PD could most likely be mediated by GPR109a as previously mentioned [33].

In addition, it was also found that BHB inhibits inflammation by promoting microglial ramification. Interestingly, our review found that BHB treatment significantly reduced the IBA-1 marker (activation marker) in microglia in both PD and AD models [33,44]. The suppression of microglial activation did not only affect activated cells, but also non-plaque-associated microglia, which were found to be ramified/inactivated in an AD mouse model fed BHB [43]. This ramification process is reversible and is likely mediated by the inhibition of the histone deacetylases (HDACs) and not GPR109A [41]. Moreover, polarization from the microglial M1 to the M2 phenotype was also found to be promoted upon the inhibition of the NLRP3 inflammasome [42].

Additionally, the regulation of the NLRP3 inflammasome has been connected to autophagy. Impaired autophagy was found to exacerbate β-amyloid-induced NLRP3 inflammasome activation and *IL-1β* release [63]. Additionally, Caspase-1 activation brought on by the β-amyloid peptide causes neurodegeneration and memory loss in AD mice by disrupting autophagy in the cortex and hippocampus [64]. Moreover, microglia-specific Atg7 deletion increases the spread of intraneuronal tau pathology and makes microglia proinflammatory in vivo and in vitro. [65]. Likewise, the prevention of a-synuclein pathology in PD by NLRP3 inflammasome inhibition was also reported to depend on improving autophagy function [60]. Despite this, BHB’s inhibitory effects on NLRP3 did not rely on autophagy [62] and, according to a recent observation, did not also involve GPR109a [66]. Interestingly, the biosynthesis of BHB, however, can be impaired by preventing autophagy [66]. This could imply that autophagy was improved or sustained in a yet-to-be-determined mechanism following BHB intervention, allowing for the reduction in plaque size and average areas occupied by plaques seen in AD mouse models [43,44].

Additionally, in human studies, the effect of BHB on NLRP3 inflammasome signaling remains controversial. Exogenous administration of ketone bodies, according to two studies, had no significant effect on inflammasome inactivation [67,68]. However, in a recent study, individuals who consumed an isocaloric KD for three days saw an increase in BHB and fibroblast growth factor-21 (FGF-21) serum levels, which significantly decreased inflammation [69]. Due to the sheer complexity of the study design, the results are mixed, implying the need for additional human studies in the near future to properly determine the effects of BHB or KD in general on the NLRP3 inflammasome.

### 4.3. BHB Effects on Cognitive/Physiological Improvements in Neurodegeneration

Furthermore, in neurodegenerative rodents, BHB was found to alleviate symptoms of cognitive decline, motor skill deterioration, and behavioral deficits. Mice treated with BHB outperformed 5XFAD mice given PBS in the spatial learning task (Morris water maze test) and retained their memories better in the hidden platform task [44]. Similar findings were made in a different study, where 3xTgAD mice fed a ketone ester (KET) diet demonstrated noticeably more exploratory behavior in the elevated plus maze test and open field test [70]. A noticeably shorter freezing time during tone-associated fear conditioning was also recorded in these mice relative to standard diet-fed mice [70]. This favourable impact of BHB was related to the suppression of microglial overactivation and the protection of the dopaminergic neurons in the substantia nigra of LPS-PD model. In the event of persistent inflammation, decreased IBA-1 levels as well as BHB’s inhibition of the activation of inflammatory mediators such the NF-kB via the GPR109a prevented damage to the dopaminergic neurons. Thus, neuron functionality is preserved, providing additional protection against motor dysfunction [33]. When evaluated for rotational behavior, BHB attenuated the amphetamine-induced rotation in these rats compared to controls [33]. Interestingly, KD pretreatment reduced the motor dysfunction (improved rota rod test) brought on by 1-methyl-4-phenyl-1,2,3,6-tetrahydropyran (MPTP) in mice, which produce molecular alterations resembling those seen in idiopathic PD patients [71]. In comparison to the standard diet MPTP group, the pretreatment improved rota-rod test results in these mice [71]. Human studies on BHB’s neurophysiological responses are still in the early stages, and there has yet to be enough data to establish its anti-inflammatory effects with convincing evidence. However, nutritional research in KD is expanding, with promising results in patients with neurodegenerative disorders. A study found that patients without the APOE-4 allele, a risk gene for sporadic Alzheimer’s disease, performed better on the Alzheimer’s Disease Assessment Scale-Cognitive Subscale (ADAS-cog) than patients with the allele after receiving treatment with medium-chain triglycerides (MCT) [72]. Greater improvements in recalling paragraphs after MCT treatment compared to placebo were associated with higher BHB values across all subjects [72].

Similar findings were observed in patients given the oral ketogenic compound AC-1202 [73]. Additionally, it has been demonstrated that mild to moderate AD patients who used a KD for two to three months had improved short- and long-term, working memory, and processing speed [74]. Additionally, those with mild cognitive impairment brought on by PD experienced an improvement in cognitive function after nutritional ketosis [75]. When compared to the high-carbohydrate-fed group, the low-carbohydrate-fed group performed better in the Controlled Oral Word Association task, particularly in the lexical access trials, and showed less interference in memory [75]. The KD group also displayed greater improvements in non-motor symptoms in comparison to the low-fat diet cohort, as was demonstrated in a different study [76]. Furthermore, a study on neuronal dysfunction discovered that KD-induced elevation of serum BHB causes improvement in forelimb motor function following SCI in rats [77], with one supporting study showing inflammation amelioration through inhibition of the NLP3 inflammasome [42]. The study also discovered improved grip strength and ipsilateral forelimb use after KD was administered [42]. These show that BHB could be a useful therapeutic tool for treating motor dysfunction caused by neuro-inflammation. The supplemental figures paper gives a succinct graphical explanation of how BHB therapy affects microglia function and/or activation (Figures S1, S2 and S3a,b).

### 4.4. Limitations of Study and Future Recommendations

Overall, the research papers gathered for this review have shown that BHB has therapeutic potential for reducing microglial inflammation in the context of neurodegenerative diseases. The review’s main limitation is that it only looked at five studies, two of which were on AD, two on PD, and one on SCI. While making a comparative evaluation within the same diseases can be difficult due to the heterogeneity in the study model; a wide range of administration and/or inducing methods of BHB, experimental animal species used, age and sex at study onset, study duration, or inflammatory stimuli, the results, however, were consistent, providing confidence in the intervention’s effectiveness. Having a fixed method of inducing endogenous conversion of triglycerides into ketones would be best for further consideration as there is still a sizable gap in the translatability of the ideal BHB dosage and treatment duration in animal studies to human applications.

Additionally, the reported works concentrate on using the familial AD model, which mainly represents a rare form of the disease that only makes up less than 5% of all AD cases. The need for sporadic model studies on the effectiveness of BHB is highly warranted and would be highly relevant in this context. To provide a more complete picture of the effectiveness of BHB in slowing the progression of neurodegeneration (early or late phase) through microglial modulation, interventions should be carried out at various time points. Furthermore, studies on the precise mechanism by which BHB regulates tauopathy should be conducted in depth. It is undeniable, however, that the development and/or use of suitable sporadic animal models of AD in the preclinical stage generally, is a significant difficulty in and of itself, given that existing models only accurately reflect partial abnormalities in sporadic pathology. The loss of neurons and the development of neurofibrillary tangles (NFTs) are two examples of this, along with the notable age, physiology, genetic, lifestyle, and/or cognitive differences between humans and other animals [78]. The challenge is far more substantial in rodents as the anti-AD effects seen are rarely replicated in humans. One possibility for bridging this gap is to create a viable AD model in nonhuman primates. These animals have similar biological characteristics to humans, including normal physiology of aging, and may be trained to do complex tasks utilized in behavioral investigations [78]. This, too, raises ethical concerns, but it is a realistic approach for furthering our understanding of the development of AD. Similarly, in this regard, animal models of Parkinson’s disease, particularly the one highlighted in this review, may have a restriction in terms of their ability to be fully translatable to humans, as this model allows for specialized research of the disease’s specific pathogenic process. In this model, stereotaxic injection of the bacterial endotoxin LPS generally activates microglia and causes dopaminergic neuronal death as well as α-synuclein protein buildup in tyrosine-hydroxylase positive neurons in the substantia nigra and striatum of animals [79]. While this model is commonly used to better understand the molecular mechanisms behind the inflammatory processes in Parkinson’s disease, it remains to be fully elucidated as to how the treatment causes selective damage to dopaminergic neurons [79]. Furthermore, because of the size of the substantia nigra and the dense dispersion of dopaminergic neurons, direct LPS treatment might induce significant neuronal harm [79]. Additionally, following the molecular changes revealed, this model demonstrated a loss in motor functions but lacked non-motor symptoms that are commonly reported in Parkinson’s disease patients [79]. The rapid progression of alterations found in this model raises concerns about properly understanding the disease’s pathophysiology. Similarly, to Alzheimer’s disease, the use of non-human primates with similar anatomical and genetic backgrounds to humans opens up new paths for more reliably studying the disease’s complicated etiology.

There has only been one study that has found a link between the ketone body and tau. According to the study, BHB can lower the amounts of tau tangle aggregates brought on by a high-fat diet in ApoE-deficient mice when compared to controls [80]. Moreover, primary cell cultures or recently isolated glial cells are better able to accurately reflect the extent of changes following stimulation with various agents than microglial cultures; BMDM, HT22, or BV-2 cells. The findings would be further validated with the use of a much more reliable model such as the human induced pluripotent stem cell-derived microglia (hiPSC-Microglia).

Additionally, a more recent study discovered that the KD alters neuroinflammation in mice with repetitive mild traumatic brain injury (rMTBI) via metabolites from the gut microbe *Lactobacillus reuteri* [81]. In these mice, treatment reduced microgliosis and neuroinflammation while improving neurological function [81]. This has enormous potential application for BHB’s ability to modulate the general gut microbiome and reduce inflammation in people with neurodegenerative diseases.

It’s interesting to note that a recent finding showed the SARS-CoV-2 virus can interact directly with microglia, causing a strong inflammasome activation [82]. In the same study, it was found that when an α-synuclein-activator-mediated inflammasome activation was present, the activation was further enhanced [82]. These findings raise the possibility that an infection could affect synucleinopathy and by extension the progression of PD. A complementary study that used bioinformatics also found a connection between SARV-COV-2 and PD through, among other things, the microglia pathogen phagocytosis pathway and microglia activation, which was suggested to have been significant in the onset or progression of PD [83]. In a similar vein, AD and COVID-19 share multiple risk factors and pathogenic pathways that may contribute to the acceleration of neurodegenerative processes in AD patients infected with the virus [84]. Recent COVID-19 studies were presented with promising benefits of KD and BHB supplementation. The administration of BHB as a ketone ester and a KD restores CD4+ T cell metabolism and function, allowing mice infected with SARS-CoV-2 to live longer [85]. Additionally, when exposed to COVID-19, beta-hydroxybutyrate increases the immune function of human T-cells and changes their metabolism to increase the activity of the mitochondrial respiratory chain, providing them with more energy [86]. These encouraging findings have yet to be translated into the framework of viral infection and the progression of neurodegenerative diseases, opening new avenues for BHB and KD intervention for the modulation of microglial inflammation.

Finally, a window of opportunity exists for the treatment of neurodegeneration by targeting the metabolism of the microglia. A recent investigation revealed that β-amyloid induces NLRP3 inflammasome activation by influencing microglial immunometabolism via the Syk-AMPK pathway [87]. Additionally, BHB was discovered to facilitate LPS-induced glycolytic intermediate accumulation and, paradoxically, promoted the upregulation of pro-inflammatory marker genes [40]. Contrastingly, our review found that BHB enhanced hippocampal neurons’ ability to produce ATP and improved their mitochondrial respiratory function, both of which helped the neurons recover from β-amyloid toxicity and ROS injury [44]. These contradictory findings may be explained by the triggers that activated these microglia. Even so, research into how microglia’s immunometabolism contributes to neurodegeneration is still in its infancy and needs to be expanded upon. It would additionally be wise to investigate how changing the metabolism of the microglia through dietary or external administration of BHB might contribute to preventing the disease from progressing.

## 5. Conclusions

We summarized BHB’s promising potential for reducing inflammation in neurodegenerative disorders via modulating microglial function. The GPR109a receptor and the deactivation of the inflammasome are two mechanisms through which BHB functions. While it is vital to understand the processes by which this ketone body functions, given the various roles it has been linked to in recent studies, there is little doubt that the potential protective and preventative qualities of BHB are enormous, given the direct modulatory impact it has on individual microglia. No doubt clinically validated therapeutic outcomes could support BHB as a suitable, affordable tool for the preventative measures and treatment options of neurodegenerative disorders associated with chronic inflammation.

## Figures and Tables

**Table 1:** Summary of data extracted from eligible articles.

Paper	Disease Context	Findings
[43]	AD	BHB levels in the brain parenchyma and blood are lower in AD patients compared to healthy controls. Β-amyloid plaques and microgliosis were found to be reduced following BHB treatment in a transgenic mice model relative to controls The proinflammatory primed state of microglia is shifted post-BHB treatment to a less complex resting morphology in the transgenic mouse model compared to controls. NLRP3 inflammasome activation was inhibited in BHB-treated BMDM cells In comparison to untreated controls, BHB treatment reduces the release of mature *Il-1β* cytokine in transgenic mice. No cognitive tests were conducted.
[44]	AD	In comparison to saline-treated control mice, BHB treatment reduces the number of amyloid plaques in the transgenic mice model of AD. In comparison to untreated transgenic mice, the amount of soluble β-amyloid-40 and β-amyloid-42 in hippocampal and cortical brain homogenates decreased significantly after BHB treatment. BHB-treated mice exhibited reduced IBA-1-positive microglia compared with saline-treated controls. Genetic expression of *Il-6*, *Tnf- α* and *Il-1β*, was significantly lower in both cortex and hippocampus of BHB-treated mice than in saline-treated controls. In a GPR109A-dependent manner, BHB treatment inhibits partial APP processing while increasing NEP expression. The Morris Water Maze test revealed that BHB-treated mice outperformed saline-treated controls.
[45]	PD	BHB treatment does not prevent fibrillar synuclein aggregate-induced primary microglial activation compared to ATP and monosodium urate (MSU).
[33]	PD	Compared to controls, BHB treatment improved motor dysfunction in PD rats that were subjected to LPS. Tyrosine hydroxylase was expressed more frequently, and more cells were positive for it after BHB treatment in the LPS-induced PD model. After receiving BHB treatment, LPS-injected rats exhibit a dose-dependent shift in the microglia’s proinflammatory primed state. The expression of *Cox-2*, *Il-1β*, *iNOS*, and *Tnf- α* was found to be downregulated after BHB treatment in the model. In BHB-treated rats, GPR109A inhibits LPS-induced production of COX-2, IL-1β, iNOS, and TNF-α proteins. BHB treatment causes a reduction in p65 (NF-kB) levels following LPS stimulation in primary microglial cultures and is GPR109A-dependent.
[42]	SCI	Following spinal cord injury, intravenous administration of BHB reduced the levels of CD11b, IL-1β, and TNF- α proteins. In LPS + ATP co-treated BV2 cells, BHB pretreatment dose-dependently decreased CD11b, IL-1β, and TNF- α proteins. BHB pretreatment of BV2 cells resulted in dose-dependent reductions of ASC, NLRP3, and Caspase-1 p20 levels that were significant The proinflammatory primed state of microglia is shifted to an M2 state by decreasing CD16 protein levels and increasing Arginase 1.

AD; Alzheimer’s Disease, PD; Parkinson’s Disease, SCI; Spinal Cord Injury, NLRP3; NLR family, pyrin protein domain 3, BMDM; Bone marrow-derived macrophages, ASC; Apoptosis-associated speck-like protein containing a caspase recruitment domain.

## Data Availability

All data generated or analyzed during this study are included in this published article.

## Supplementary Materials

The following supporting information can be downloaded at: https://www.mdpi.com/article/10.3390/nu15030524/s1, Figure S1: The role of different microglial phenotypes; Figure S2: The influence of BHB on the activation of the NLRP3 inflammasome and GPR109A; Figure S3a: Neuronal degradation in PD, AD, and SCI; Figure S3b: BHB’s neuroprotective effects on degenerating neurons.
