# Supplementary material for: β-Hydroxybutyrate Regulates Activated Microglia to Alleviate Neurodegenerative Processes in Neurological Diseases: A Scoping Review
Source: Nutrients. 2023 Jan 19;15(3):524. doi: 10.3390/nu15030524 (PMC9921456; doi:10.3390/nu15030524)
Supplement: Supplementary file 1 [file nutrients-15-00524-s001.zip › nutrients-2083870-supplementary.pdf]

Supplementary Materials

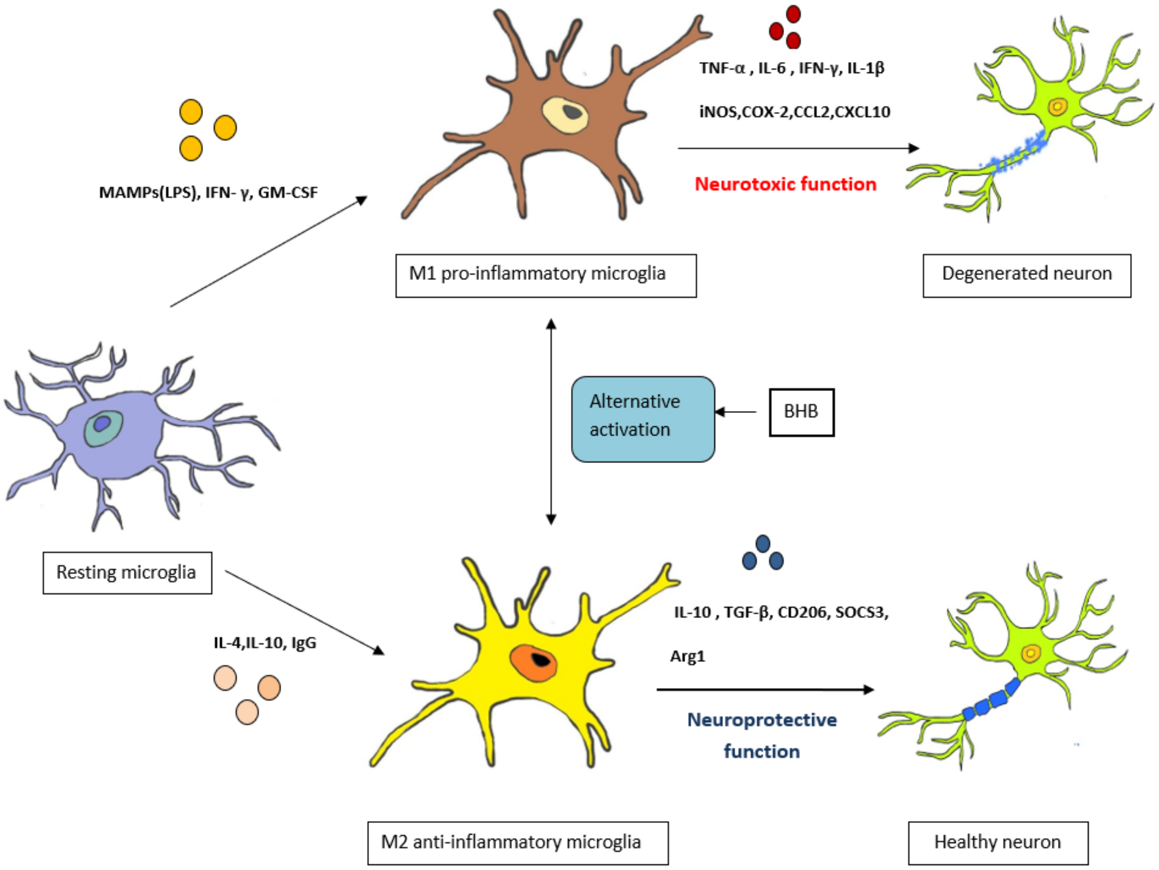

Figure S1. The role of different microglial phenotypes.

Microglia are classically known to undergo 2 types of activation based on their stimulant. Stimulants like LPS, IFN-γ, and GM-CSF instigate activation of the M1 microglia phenotype, which undergoes morphological changes, converting the resting microglia into an amoeboid-shaped pro-inflammatory state. The M1 phenotype promotes the activity of pro-inflammatory responses through chemokine like CCL2 which allows for the activation of CXCL10 expression found in microglia. This mechanism initiates the production of pro-inflammatory cytokines and mediators like TNF-α, IL-6, IFN-γ, IL-1β,iNOS, and COX-2 which could facilitate in eliminating the pathogens and damage-causing proteins. While this process is beneficial, chronic activation of microglia in its M1 phenotype in a degenerative disease tends to aggravate the release of pro-inflammatory cytokines and mediators which eventually becomes neurotoxic towards any neuronal structure and function, creating degenerated neurons. Meanwhile, some stimulating molecules like IL-4, IL-10, and IgG activate the resting microglia to M2 anti-inflammatory phenotype. The M2 phenotype increases the expression of microglia surface marker CD206 and inflammatory cytokines, mediators, and signaling proteins such as IL-10, TGF-β, Arg1, and SOCS3 that possess the neuroprotective function in neurons. Thus, neurons do not undergo damage and are protected from the anti-inflammatory properties of M2

microglia. Alternatively, M1 and M2 microglia can alternate between their activation states depending on the environmental factors that regulate their function. BHB is known to be able to reduce its pro-inflammatory responses while increasing Arg1, thus switching the M1 phenotype to M2 microglia.

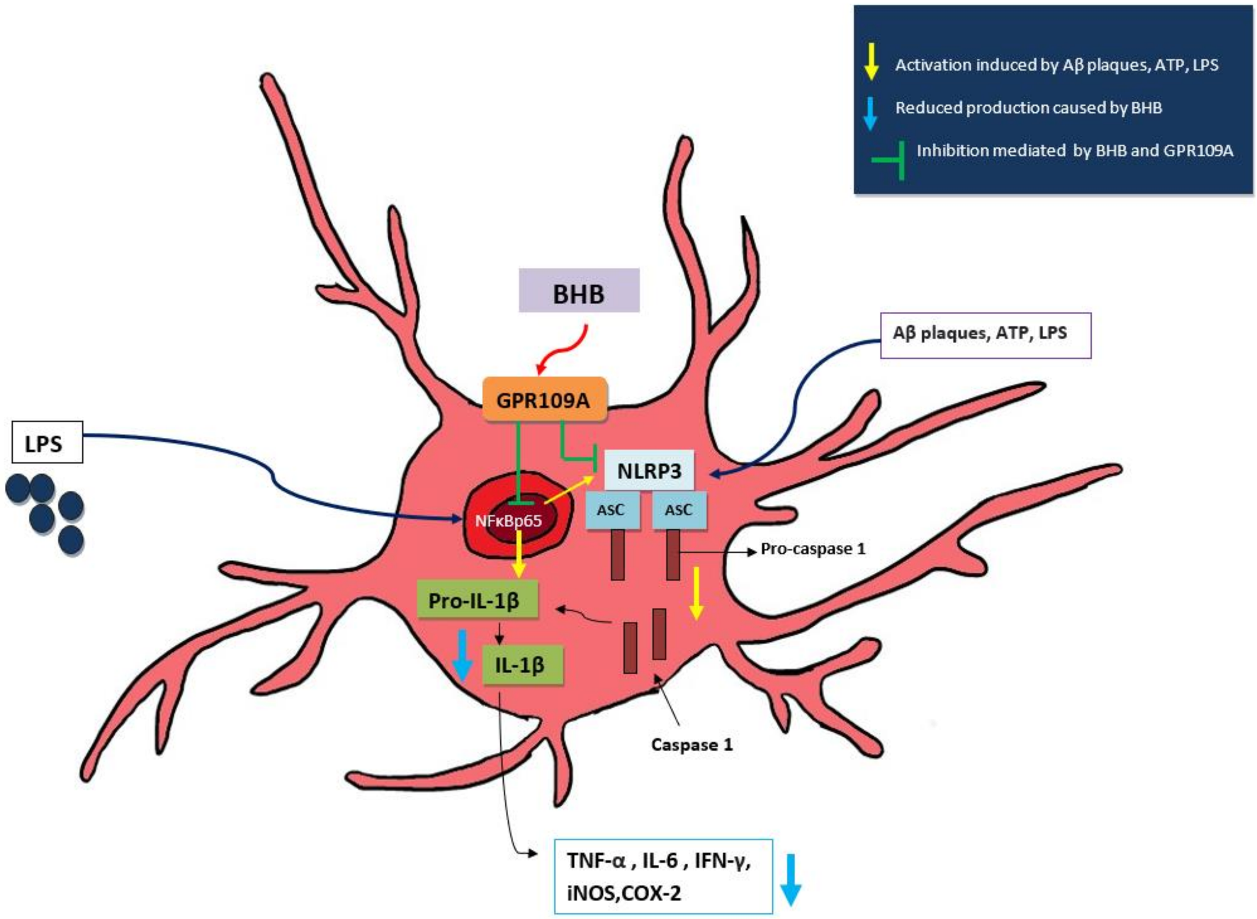

Figure S2. The influence of BHB on the activation of the NLRP3 inflammasome and GPR109A

The NLRP3 inflammasome activation requires 2 steps: priming and activation. The priming step is initiated by TLR4 agonists like LPS, which mediates NFkB expression to stimulate NLRP3 and *pro-Il-1β* expressions as well, followed by the activation part that is triggered by MAMPs, PAMPs like Aβ plaques and LPS, and also ATP. This leads the ASC to aggregate and assemble into a more complex protein called ASC specks. The inflammasome assembly activates the conversion of pro-caspase-1 into caspase-1, which functions in cleaving the pro-inflammatory cytokine pro-IL -1β to a matured molecule IL -1β.

As a key mediator of the inflammatory response, IL-1β promotes host response and resistance to foreign bodies and pathogens by releasing other pro-inflammatory cytokines and enzymes like TNF-α, IL-6, IFN-γ, iNOS, and COX-2. In the event of chronic pathogenesis in degenerative diseases like Alzheimer's disease and Parkinson's disease, the inflammatory reaction is exacerbated, thus damaging more neurons instead of protecting them. Hence, neuronal functions are

deteriorated, developing symptoms like poor cognitive and motor function as seen in the mentioned diseases.

BHB binds to GPR109A to inhibit the transcription of p65-associated NFkB and its mediated priming of NLRP3 activation. Besides that, BHB can directly prevent the activation of NLRP3 inflammasome via GPR109A manner. Therefore, there is reduced production of caspase-1 and IL -1β to contribute to pro-inflammatory responses. This eventually results in a lower level of pro-inflammatory cytokine and enzymes, compromised potential for APP processing in β-amyloid plaque aggregation, and increased enzyme like NEP for cleavage of Beta-amyloid proteins. Therefore, BHB is seen as a metabolite that is capable to restore the function of healthy neurons.

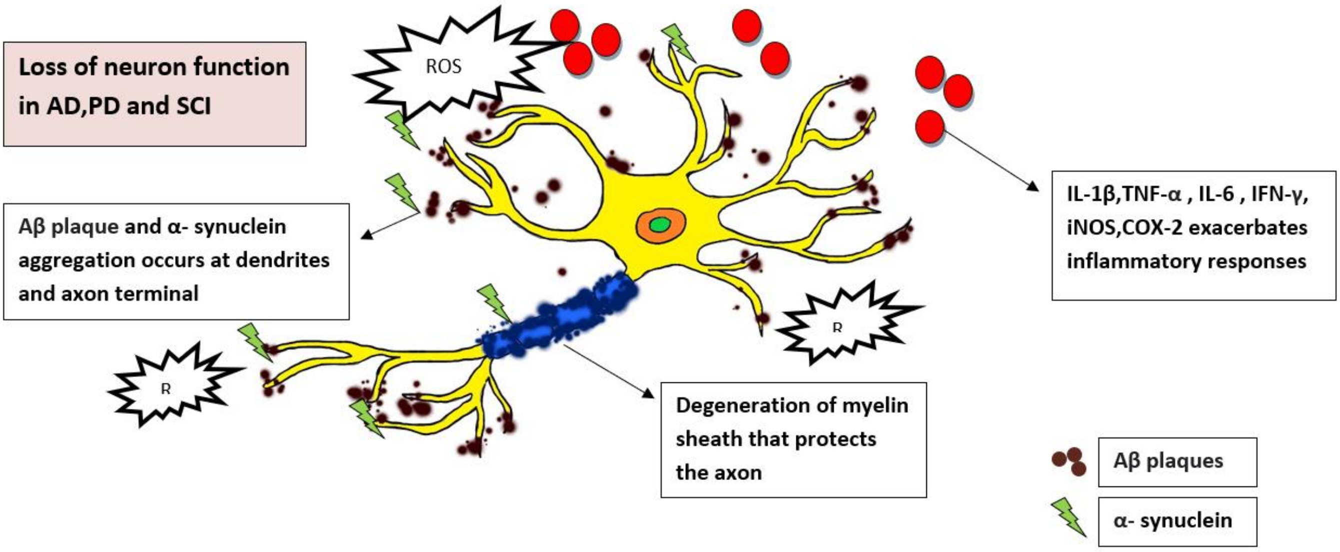

Figure S3a. Neuronal degradation in PD, AD, and SCI

Neuron disruption is a crucial feature in neurodegenerative disease progression. Damage-causing proteins like Aβ plaques in AD and α- synuclein in PD aggregate at various parts of neurons such as the dendrites, axons, and axon terminals. They tend to induce oxidative stress in neurons and subsequently enhance the production of ROS. Similarly, in the secondary injury phase of SCI, increased ROS and reduced GPx, GSH, SOD, and CAT worsen neuron protection. Moreover, Aβ plaques, α- synuclein, and ROS are prominent triggers for microglia activation into its M1 pro-inflammatory state, where prolonged activation leads to a condition known as microgliosis. Microgliosis intensifies the inflammatory activity of the pro-inflammatory cytokines and mediators like IL-1β, TNF-α, IL-6, IFN-γ, iNOS, and COX-2, which augments the degeneration process in neurons. Gradually, the neuron loses its ability to transmit signals at a better speed, exchange signal with another neuron, or promote synaptic activities. Hence, the structural deterioration encountered in the neuron is followed by the impaired function that it carries. This, therefore, explains symptoms or signs like cognitive impairment or motor dysfunction that commonly predominates in the pathogenesis of AD, PD, or SCI.

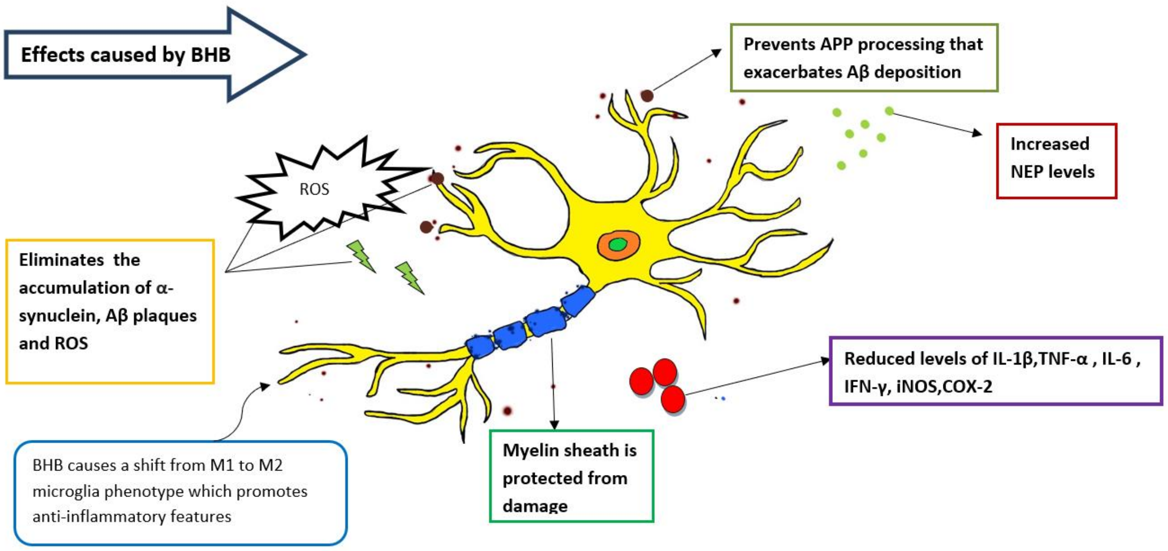

Figure S3b. BHB's neuroprotective effects on degenerating neurons.

This figure describes the effects shown by BHB in preventing neuron degeneration caused by inflammation in the case of AD, PD, and SCI. BHB increases the level of amyloid-degrading enzyme neprilysin (NEP) and prevents APP processing for β-amyloid peptide formation, hence resulting in a total reduction of Aβ plaques in the AD disease models. BHB treatment also causes shifting of M1 microglia to resting microglia or M2 phenotype, subsequently lowering the levels of pro-inflammatory cytokines and mediators produced, meanwhile increasing anti-inflammatory markers like Arg1. Thus, alleviated inflammatory responses allow for lesser oxidative stress faced by neurons, which inhibits ROS aggravation. Furthermore, these effects restore the function and structure of a neuron, including the recovery of myelin sheath from deterioration. Eventually, cognitive ability, behavior skills, and motor functions oversee a positive outcome from BHB treatment in AD, PD, and SCI.
